# *In vitro* priming with *Mycobacterium fortuitum* induces trained immunity-associated phenotypes in caprine alveolar macrophages and enhances phagosomal maturation and antimicrobial functions upon homologous re-stimulation

**DOI:** 10.3389/fcimb.2026.1927556

**Published:** 2026-08-26

**Authors:** Miriam Blay-Benach, Patricia Cuenca-Lara, Julia Moraleda, Zoraida Cervera, Joan Repullés, Bernat Pérez de Val

**Affiliations:** 1 IRTA, Programa de Sanitat Animal, Centre de Recerca en Sanitat Animal (CReSA), Campus UAB, Bellaterra (Cerdanyola del Vallès), Spain; 2 Unitat mixta d’Investigació IRTA-UAB en Sanitat Animal, Centre de Recerca en Sanitat Animal (CReSA), Campus Universitat Autònoma de Barcelona (UAB), Bellaterra Catalonia, Spain

**Keywords:** alveolar macrophages, bactericidal responses, goat, iNOS, macrophage activation, *Mycobacterium fortuitum*, phagocytosis, trained immunity

## Abstract

**Background:**

Trained immunity refers to the stimuli-induced epigenetic and metabolic reprogramming of innate immune cells, enabling enhanced non-specific responses upon secondary stimulation. Alveolar macrophages (AMs) constitute the primary immune barrier in the lung and represent a key target in enhancing protection against pulmonary pathogens.

**Methods:**

In this study, an *in vitro* priming-resting-re-stimulation protocol was used to characterise the ability of *Mycobacterium fortuitum* (MF) to induce functional features of trained immunity in caprine AMs *in vitro*. Pro-inflammatory cytokines (TNFα, IL-1β, IL-6) and nitric oxide synthase (iNOS)-positive cells were quantified by multiplex assay and flow cytometry, respectively. Phagosomal acidification was detected by flow cytometry and live-cell fluorescent microscopy after MF-challenge using pH-sensitive labelled bacteria.

**Results:**

MF-trained AMs displayed significantly elevated TNFα and IL-1β production and a higher frequency of inducible iNOS-producing cells compared to non-stimulated controls, consistent with a trained immunity-associated pro-inflammatory and antimicrobial phenotype. Phagosomal acidification was enhanced and sustained in MF-trained AMs following homologous challenge with MF, suggesting enhanced phagocytic activity against mycobacteria.

**Conclusion:**

These findings provide evidence that *M. fortuitum* induces trained immunity-associated functional responses in caprine AMs and support further trained immunity studies in the caprine lung mucosa against pulmonary infections.

## 1 Introduction

Mycobacterial infections pose a significant challenge to livestock health worldwide, especially in ruminants, where both tuberculous and non-tuberculous mycobacteria (NTM) can cause substantial mortality and economic losses (WOAH, 2025; Reil et al., 2023; Pavlik et al., 2022). *Mycobacterium fortuitum* is a fast-growing environmental mycobacterium that, while not a strict pathogen, can act opportunistically, with sporadic cases reported in the veterinary field, involving mastitis in cattle (Cvetnić et al., 2022), skin lesions in cats (Malik et al., 2000), pulmonary infection in dogs (Leissinger et al., 2015), and even lymph node lesions in pigs and cattle (Kaczmarkowska et al., 2022; Nuru et al., 2017). In humans, however, infections with *M. fortuitum* are typically linked to skin and soft tissue lesions and rarely involve respiratory infections (d’Incau et al., 2020; Forbes et al., 2018; Muthusami et al., 2004). Its low pathogenicity and environmental prevalence make it a valuable model organism for studying mycobacterial biology. Understanding how such exposure shapes the innate immune response is essential for developing effective control strategies that may require novel approaches to enhance resistance against mycobacteria and other unrelated pathogens.

Innate immune cells have traditionally been considered incapable of retaining the memory traits associated with adaptive immunity. However, the emergence of the concept coined trained immunity has demonstrated that these cells can acquire a non-specific memory-like response following exposure to an immunostimulant, vaccine or strain and upon a subsequent re-stimulation (Netea et al., 2011, Netea et al., 2016, Netea et al., 2020). This process involves an epigenetic and metabolic reprogramming that leads to histone methylation and acetylation, as well as increased glycolytic activity (Cheng et al., 2014; Hole et al., 2019; Saeed et al., 2014). These changes can enhance pro-inflammatory cytokine production and the bactericidal functions of the cells, thereby strengthening non-specific host protection (Zhao et al., 2019; Quintin et al., 2012). Trained immunity reprogramming has been documented across multiple innate immune cells, including macrophages (Jeyanathan et al., 2022), monocytes (Kleinnijenhuis et al., 2012), natural killer (NK) (Kleinnijenhuis et al., 2014) and dendritic cells (Tepale-Segura et al., 2024). Some studies have shown that BCG and β-glucan stimulation can provide cross-protective effects against different unrelated pathogens or diseases (Quintin et al., 2012; van Puffelen et al., 2020; Arts et al., 2018; Hetland et al., 2000).

Alveolar macrophages (AMs) are the most predominant immune cells of the alveoli, where they serve as the first line of defence against certain inhaled pathogens, preserving pulmonary function and maintaining lung homeostasis (Malainou et al., 2023; Hou et al., 2021). Enhancing their functional non-specific responsiveness through trained immunity could improve host resistance, particularly in species that are constantly exposed to environmental pathogens. Targeted activation of AMs is especially relevant in caprine tuberculosis research, where improving bactericidal capacity and inducing an effective trained state, characterised by elevated production of the inducible nitric oxide synthase (iNOS) and expression of pro-inflammatory cytokines, may contribute to improving goats’ resistance to subsequent infections (Netea et al., 2011; Kleinnijenhuis et al., 2012; Mata et al., 2021; Guerra-Maupome et al., 2019). Despite increasing evidence in human and murine models (Jeyanathan et al., 2022; Kleinnijenhuis et al., 2012; Mata et al., 2021; Bekkering et al., 2016), little is known about trained immunity in caprine AMs or how exposure to NTM, such as *M. fortuitum*, might influence this trained immunity-associated functional reprogramming.

In the present study, we investigated whether *M. fortuitum* could induce innate immune memory effects *in vitro* in caprine AMs. To address this, macrophages were stimulated with *M. fortuitum* to assess heightened pro-inflammatory cytokine responses, increased iNOS activation, and improved phagocytic acidification upon a homologous secondary challenge.

## 2 Materials and methods

### 2.1 Reagents and *M. fortuitum* strain

AMs were cultured with complete RPMI cell medium, which contained RPMI 1640 with 10% SBF, 1% Glutamine, 1% Streptomycin/Penicillin, and 0.5% Nystatin. For stimulation and challenge, *Mycobacterium fortuitum* strain ATCC 6841 was used, which was preserved in BHI + 20% glycerol and stored at -80%.

### 2.2 Ethical declaration

One healthy goat was obtained at the Servei de Granges i Camps Experimentals (SGCE) of the Autonomous University of Barcelona, a certified TB-free farm with no history of tuberculosis, with registration number B9900042. Euthanasia for lung removal procedure was approved on 19 June 2024 by the Animal Welfare Committee of the Autonomous University of Barcelona (No. 5482-CEEAH-UAB) and the *Generalitat de Catalunya* (Reference No. 12164), in accordance with regulations on the protection of experimental animals of the European Union.

### 2.3 Alveolar macrophages isolation from post-mortem bronchoalveolar lavage

Samples for this study came from the lung of a two-month-old female goat. BAL was performed as described by Cuenca-Lara et al. (Cuenca-Lara et al., 2026) to isolate AMs. For lung obtention, euthanasia was administered by intravenous injection of pentobarbital at 200mg/kg and the trachea was carefully tied to avoid the entrance of ruminal content into the lung. Once isolated, cells were counted using Trypan Blue and cultured in a T25 flask for 24h to assess contamination and morphology. Lastly, AMs were cultured in the desired plates and concentration depending on the experimental design.

### 2.4 Experimental design

**Figure 1:**
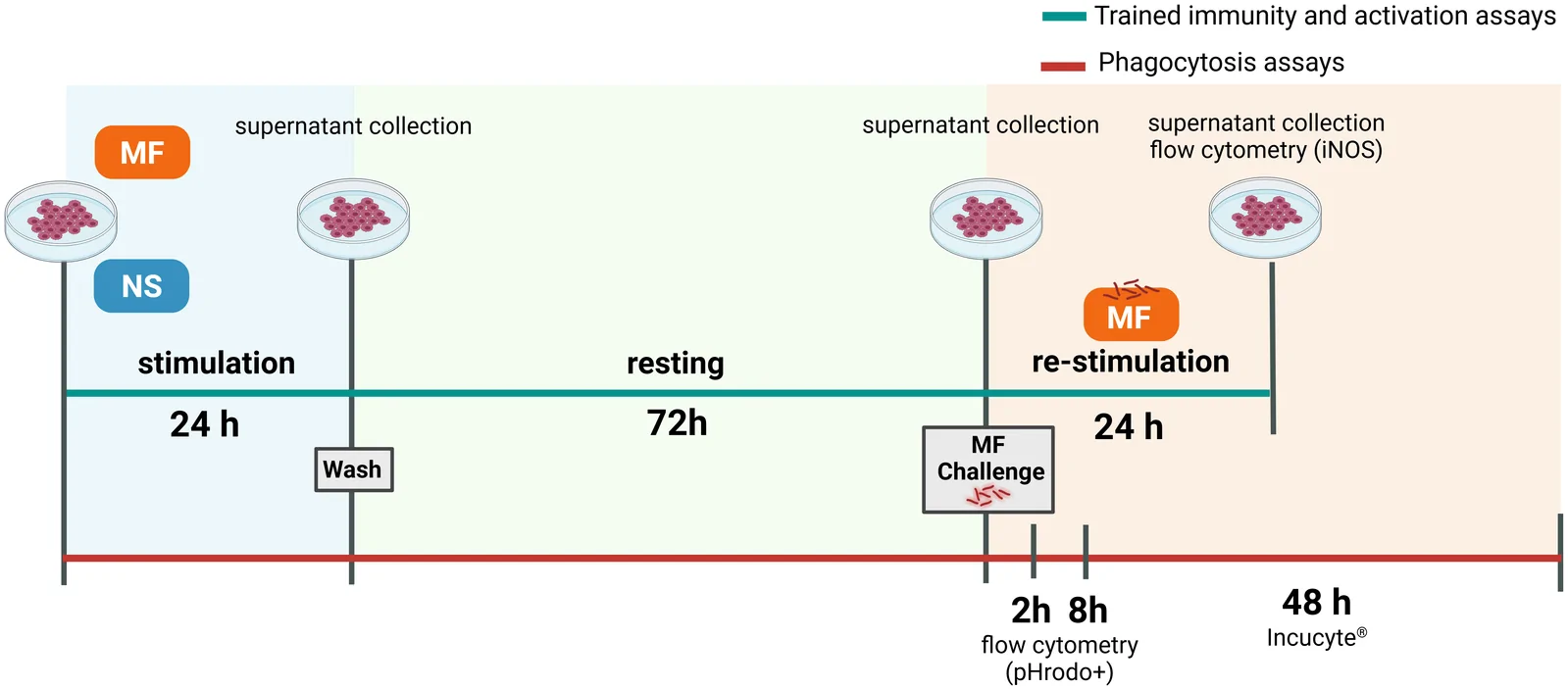
Study design. AMs were plated and stimulated for 24h with *M. fortuitum* (MF) or maintained non-stimulated (NS). After stimulation, supernatants (SN) were collected, and cells were washed and maintained in cRPMI for 72h. After the resting period, supernatants were collected and cells re-stimulated with MF. For trained immunity and activation assays, AMs were re-stimulated for 24h. After that, SN were collected and cells analysed by flow cytometry for iNOS expression detection. For phagocytosis assays, pH-dependent labelled MF was used to challenge AMs. For the flow cytometry assay, cells were either incubated for 2h or 8h and analysed for fluorescent signal. For the live-cell phagocytosis assay, cells were incubated and imaged over 48h using the Incucyte^®^ SX5 technology. Created in https://BioRender.com.

The study design is outlined in **Figure 1**. AMs were either left unstimulated (non-stimulated; NS) or stimulated with *M. fortuitum* (MF) at a multiplicity of infection (MOI) of 5 for 24h at 37°C 5% CO_2_. After 24h, supernatants were carefully collected and stored at -20°C for further analysis. A wash step was performed by adding fresh culture media to all wells, and cells were subsequently rested for 72h under the same conditions. Following the resting period, supernatants were collected and stored at -20°C for further analysis. Cells were then re-stimulated with *M. fortuitum* (same strain) at varying MOIs depending on the assay. For cytokine quantification and iNOS detection, cells were exposed to an MOI 5 of *M. fortuitum* for 24h before supernatant collection and cell analysis. For phagocytosis assays, pH-dependent labelled mycobacteria were used. A flow cytometry assay was performed using MOI 3, with cells analysed at 2h and 8h post-infection. A live-cell fluorescence imaging assay was conducted at MOI 20, with phagosome maturation monitored for 48h. All experiments were performed using technical replicates derived from AMs isolated from a single goat.

### 2.5 Pro-inflammatory cytokine quantification

Supernatants were obtained at different time points, reflecting different accumulation intervals rather than continuous kinetics: 24h after stimulation, 96h after stimulation (72h of resting) and 24h after re-stimulation (120h after priming) in the experimental workflow to assess the evolution of cytokine production of the cells. First 10^6^ cells/well were stimulated with MOI 5 of *M. fortuitum* or maintained with cRPMI in a 24-well plate and incubated at 37°C with 5% CO for 24 h. After that, supernatants were collected, and 1ml was added to all wells to rest the cells for 72h at 37°C with 5% CO_2_. Following, supernatants were collected, and all cells were re-stimulated with *M. fortuitum* at MOI 5 for 24h under the same conditions. Supernatants were collected again at the end of the experiment. Three pro-inflammatory cytokines (IL-6, IL-1β, TNFα) were analysed using a custom-made bovine multiplex kit (MILLIPLEX^®^ Millipore, Merck Life Science, Darmstadt, Germany) following the manufacturer’s instructions. Plate reading was performed using the MAGPIX system with xPONENT software (Version 4.3) (Thermofisher Scientific, Waltham, MA, USA).

### 2.6 iNOS expression

For quantification of iNOS-producing cells, 10^6^ AMs/well were stimulated for 24h with MOI 5 of *M. fortuitum* or maintained non-stimulated. Following, cell supernatants were removed, and 1ml of fresh media was added, cells were then incubated for 72h at 37°C with 5% CO2. Lastly, supernatants were removed, and cells were re-stimulated with *M. fortuitum* at MOI of 5 for 24h under the same conditions. After 6h of re-stimulation.

Brefeldin A was added to the wells to a final concentration of 10µg/ml to block the intracellular protein trafficking overnight. The following day, supernatants were removed, and cells were detached from plates by adding 200 μL of PBS for 20 minutes at room temperature (RT). Detached cells were carefully transferred to a 96-well round-bottom plate. First, cells were extracellularly stained with a viability marker (Viobility 405/520 fixable dye, Miltenyi Biotech, Bergisch Gladbach, Germany). For intracellular iNOS staining, cells were first permeabilised with Leucoperm and stained for 20 minutes at 4°C in darkness with a mouse iNOS antibody that was conjugated with Alexa fluor 647 (Clone, 4E5) (Thermofisher Scientific). Lastly, stained cells were fixed with 1% paraformaldehyde and read using the MACSQuantify™ instrument (Miltenyi Biotec). The gating strategy and the viability data is shown in the **Supplementary Figures 1**, **2**.

### 2.7 Phagocytosis assays

#### 2.7.1 Phagocytosis of pH-sensitive labelled *M. fortuitum* analysed by Incucyte^®^ SX5 live-cell system

This protocol was adapted from Blay-Benach et al. (2026) (Blay-Benach et al., 2026), with centrifugations adjusted to 3200g for 15 minutes and using the pHrodo^®^ Cell Labeling Kit for Incucyte^®^ Phagocytosis Assays (No:4766, Sartorius, Göttingen, Germany). For infection, 5 x 10^4^ AMs per condition, following the stimulation scheme (**Figure 1**), were plated in three different replicates and challenged at an MOI 20 before entering the Incucyte^®^ SX5Live-Cell Analysis System. Macrophages were imaged every 30 minutes for 48 hours. Image analysis was performed using the integrated AI Cell Health software module (Version 2022B Rev2), which enabled cell segmentation and extraction of single-cell mean fluorescence intensity values. From these measurements, the average orange mean intensity (OCU), corresponding to the pHrodo^®^ signal and indicative of phagosomal acidification, was calculated over time for each condition. To define highly acidifying cells, a threshold was established based on the maximum OCU value observed in control cells (CC) (OCU = 0.3). For quantitative comparison, the area under the curve (AUC) was calculated for both the average fluorescence intensity and the number of high pHrodo^®^ cells. In both cases, the minimum value among all infected conditions was used as baseline (OCU = 0.883 for intensity; 246.3 for high pHrodo^®^ cell counts).

#### 2.7.2 Phagocytosis of pH-sensitive labelled *M. fortuitum* analysed by flow cytometry.

*M. fortuitum* was labelled using the pHrodo™ Deep Red Mammalian and Bacterial Cell Labelling Kit (Ref. P35357, Thermofisher Scientific), following the manufacturer’s protocol with two modifications: centrifugation was performed at 3220g for 15 minutes at and the labelling incubation was extended for 2h at RT. Following the resting period, 2.5 x 10^5^ cells/well for each condition were plated in triplicate in non-treated plates and infected with labelled *M. fortuitum* at an MOI 3 for either 2h or 8h. At each time point, cells were detached using 200 μl PBS for 20 minutes at RT and transferred to a round-bottom plate. Cells were subsequently stained with a viability marker (Viobility 405/520, Miltenyi Biotec) and fixed with 1% paraformaldehyde before flow cytometry acquisition.

### 2.8 Statistical analysis.

Analysis was designed to compare the effects of *M. fortuitum* stimulation against non-stimulated AMs, as well as to assess the effects of re-stimulation with the same strain in both previously stimulated and non-stimulated samples. Statistical significances were performed on technical replicates. Results are presented as mean ± SD, or min to max. Statistical analysis was performed using GraphPad Prism (v8.3.9). Normality was assessed using the Shapiro-Wilk test, and comparisons between stimulation groups were conducted using the unpaired t-test with Welch’s correction. Comparison between MF and MF-MF groups for cytokine production and slopes for the OCU kinetics was conducted by a paired t-test and unpaired t-test, respectively.

## 3 Results

### 3.1 Re-stimulation with MF of caprine AMs enhanced pro-inflammatory cytokine production

**Figure 2:**
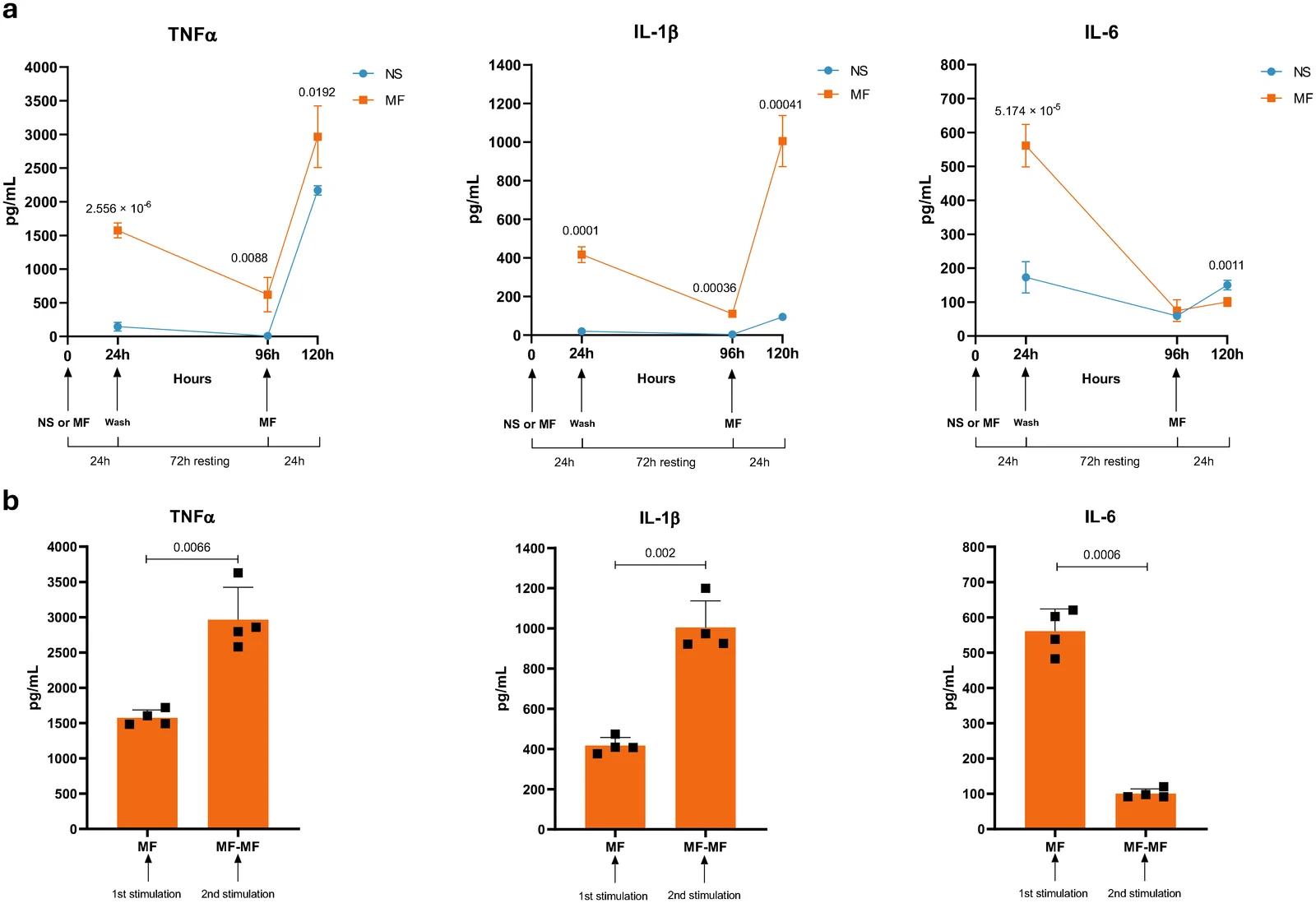
Pro-inflammatory cytokine (TNFα, IL-1β and IL-6) production by caprine alveolar macrophages. Cell supernatants were analysed at different accumulation windows: 24h after *M. fortuitum* (MF) stimulation, 72h after resting (96h after primary stimulation with MF), and 24h after MF re-stimulation (120h after primary MF-stimulation). **(a)** Graphs show cytokine concentrations (pg/mL) for non-stimulated cells (blue) and MF-stimulated cells (orange) at different time points. Data is presented as mean ± SD of four replicates. Statistical significance was determined by an unpaired t-test with Welch's correction, comparing NS vs. MF samples. **(b)** Bar graph comparisons of cell supernatants at 24h after MF prime stimulation vs. 24h after MF re-stimulation (in previously primed AMs). Data is presented as mean ± SD of four replicates. Statistical significances were determined by a paired t-test.

Cytokine production was quantified at different time points, each representing a distinct accumulation window following stimulation. Stimulating AMs for 24h with MF induced a statistically significant increase in the production of the pro-inflammatory cytokines tested, as seen in **Figure 2a**. After resting the cells for 72h with fresh cell culture media, cytokine levels were lower in both groups (MF-stimulated and NS samples). However, significant differences between the two groups were only observed for TNFα and IL-1β. Re-stimulation with MF increased TNFα and IL-1β production significantly in cells previously stimulated with MF compared to NS cells, consistent with a functional reprogramming. In contrast, IL-6 did not increase after MF re-stimulation (MF-MF group) and was slightly lower than in non-primed cells (NS-MF group). Similarly, when comparing cytokine production across time points following MF stimulation, TNFα and IL-1β production were significantly higher in double-stimulated AMs. IL-6, however, did not follow this trend (**Figure 2b**). Comparisons between stimulation regimens and time points are stated in **Supplementary Figure 3**.

### 3.2 Re-stimulation with MF increased iNOS-producing AMs

**Figure 3:**
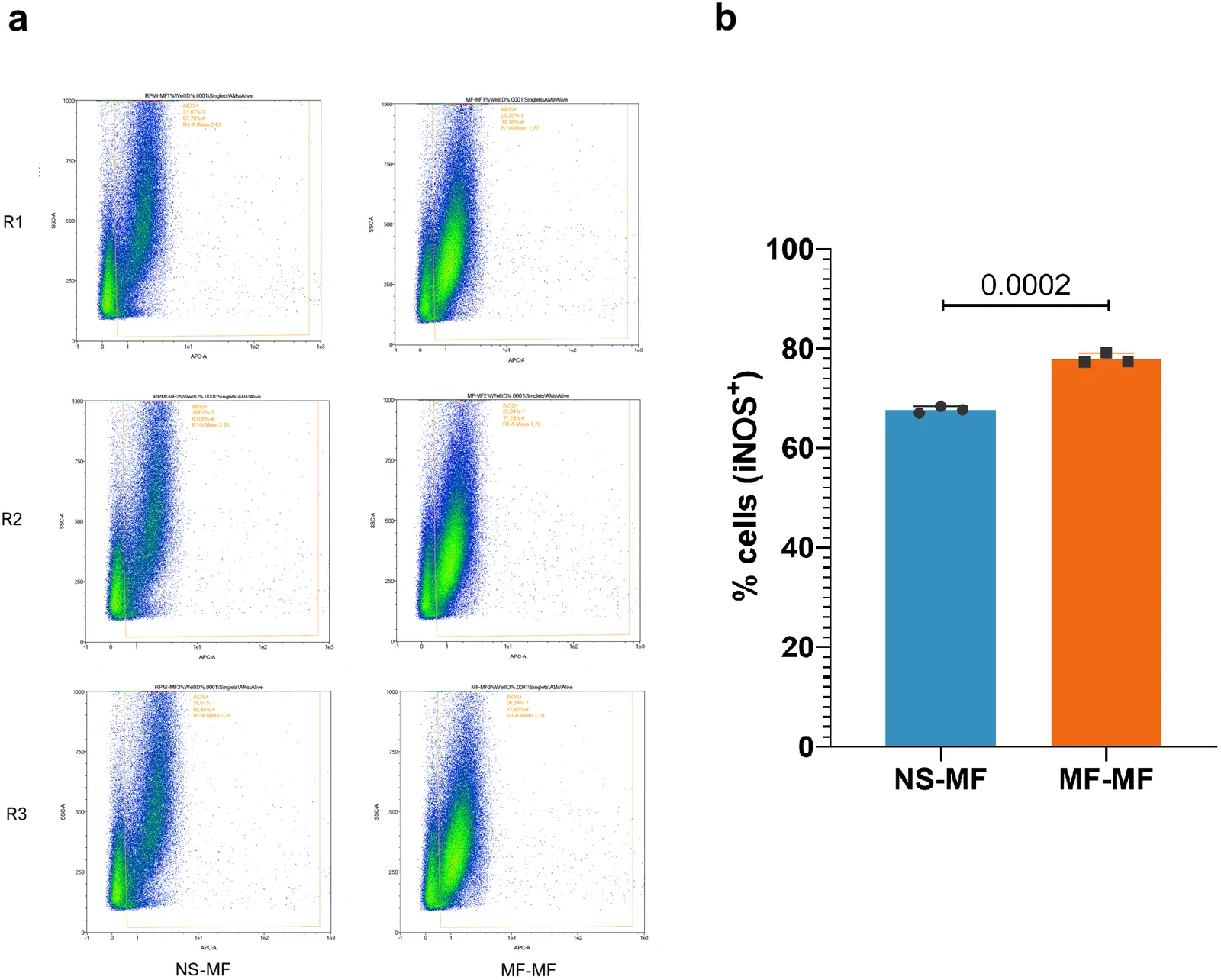
Proportion of iNOS-positive AMs after re-stimulation with *M. fortuitum* detected by flow cytometry. **(a)** Gating strategy for the three different replicates of each condition. **(b)** Bar graphs represent the percentage of iNOS-positive AMs in non-stimulated cells (NS) later exposed to (NS-MF, blue) versus MF-stimulated cells re-stimulated with MF (MF-MF, orange). Data is shown as mean + SD of three replicates. Statistical significance was calculated using an unpaired t-test with Welch’s correction.

The frequencies of iNOS-producing AMs (iNOS+) were measured by flow cytometry at 120h after stimulation (96h after priming and 24h after re-stimulation with MF). The results are shown in **Figure 3**. While both NS-MF and MF-MF groups exhibited robust iNOS expression (>60% iNOS+ cells), the MF-MF group showed a significantly higher proportion of iNOS+ cells compared to the NS-MF group (p=0.0002), suggesting functional reprogramming toward a pro-inflammatory activated phenotype following the priming-resting-re-stimulation sequence.

### 3.3 MF stimulation improved *in vitro* phagosomal acidification

#### 3.3.1 Live-cell phagocytosis assay

**Figure 4:**
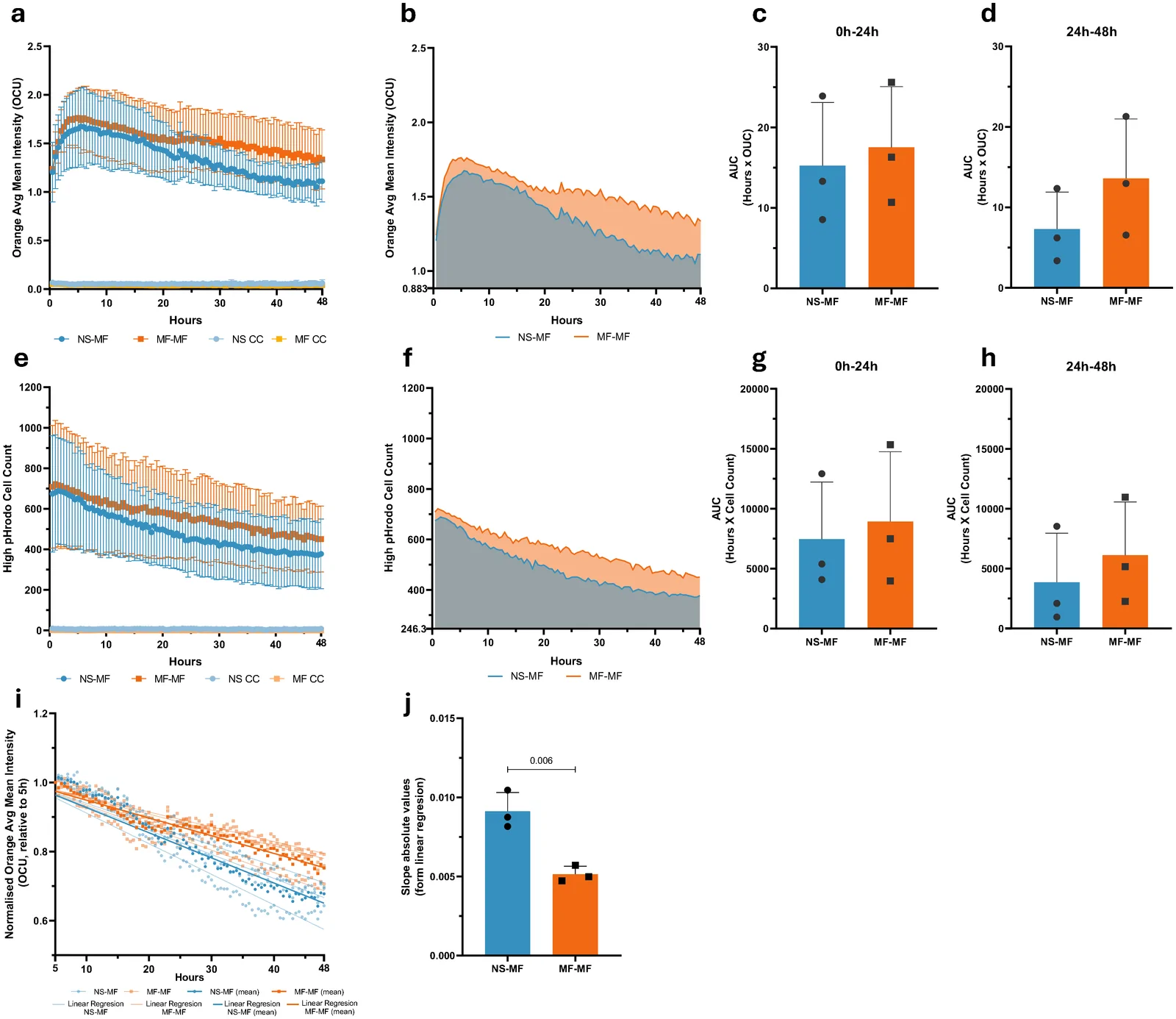
Intracellular acidification of pHrodo-labelled *M. fortuitum* in NS and MF-stimulated macrophages by live-cell imaging. **(a)** Mean fluorescence intensity (OCU) of pHrodo signal monitored over 48h in NS (blue), MF-stimulated (orange) cells and their non-infected counterparts (lighter colour variants). Data is shown as mean ± SD of three replicates. **(b)** Mean area under the curve (AUC) of the OCU kinetics, using the minimum OCU value (0.883) as baseline. **(c)** AUC (Hours x OCU) from 0–24 h, comparison between NS and MF-stimulated cells (Unpaired t-test with Welch’s correction, n=3). **(d)** AUC (Hours x OCU) from 24-48h, comparison between NS and MF-stimulated cells (Unpaired t-test with Welch’s correction, n=3). **(e)** High pHrodo-positive cell counts monitored over 48h in NS (blue), MF-stimulated (orange) cells and their non-infected counterparts (lighter colour variants). Data is shown as mean ± SD of three replicates. **(f)** Mean AUC of High-pHrodo-positive cell kinetics, using the minimum cell count (246.3) as baseline. **(g)** AUC (Hours x Cell Count) from 0-24h, comparison between NS and MF-stimulated cells (Unpaired t-test with Welch’s correction, n=3). **(h)** AUC (Hours x Cell Count) from 24-48h, comparison between NS and MF-stimulated cells (Unpaired t-test with Welch’s correction, n=3). **(i)** Linear regression of OCU values normalised to 5h. **(j)** Slope absolute values from linear regression replicates (Unpaired t-test, n=3). CC: control cells (non-infected).

Phagocytosis was evaluated at the phagosome maturation stage (acidification from pH ~7 to ~4.5) in NS and MF-stimulated cells during 48h after *in vitro* challenge with pHrodo-labelled *M. fortuitum*. Both groups exhibited similar acidification kinetics, with a peak in mean fluorescence intensity (OCU) at ~5h followed by a decline. However, the decrease was less pronounced in MF-stimulated cells (**Figure 4a**). The area under the curve (AUC; **Figure 4b**) showed that MF-stimulated cells sustained acidification over time to a greater extent than NS cells, particularly from 24h after challenge, although this difference did not reach statistical significance (**Figures 4c, d**). Analysis of the number of high pHrodo-positive cells revealed a progressive decline in both groups, with MF-stimulated cells maintaining higher cell counts over time (**Figure 4e**). Consistently, the AUC derived from this parameter showed similar trends in the MF-stimulated group, specifically from 24h hours after challenge (**Figures 4f–h**). Finally, linear regression analyses were performed on the OCU-value curves, from the peak at 5h post-challenge until the end of the experiment (48h) (**Figure 4i**). Slopes obtained for each replicate showed a higher decline in NS cells compared to MF-stimulated cells, as observed in **Figure 4j**.

#### 3.3.2 Flow cytometry phagocytosis assay

**Figure 5:**
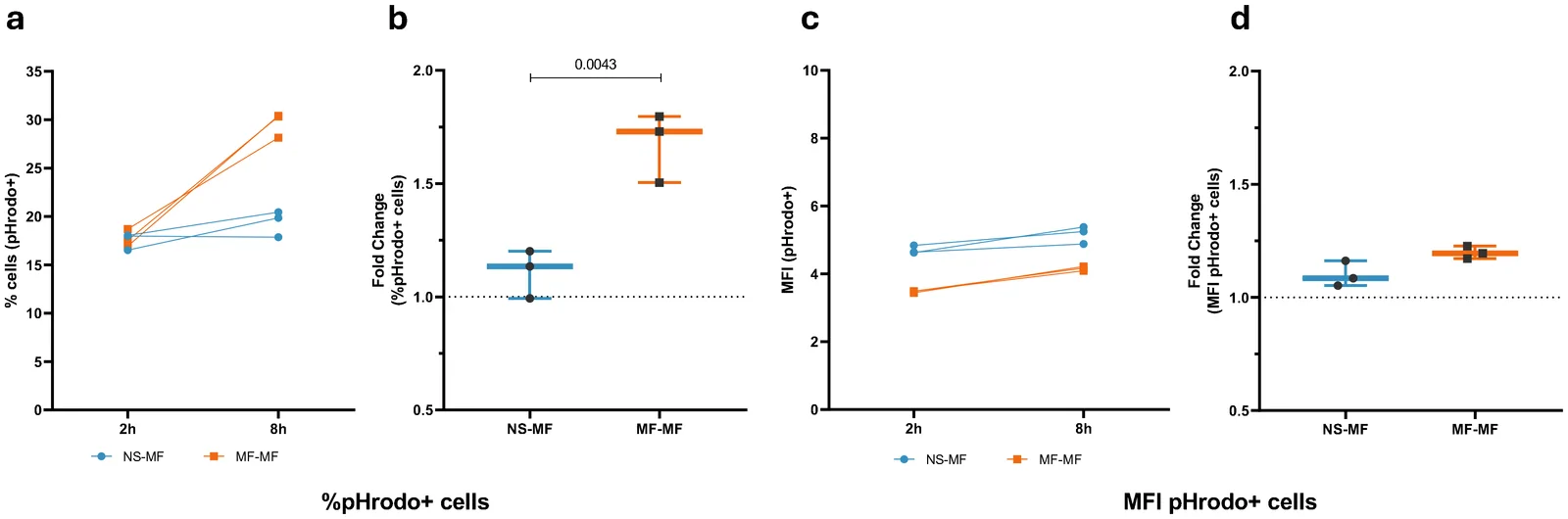
Early detection of pHrodo-labelled *M. fortuitum* signal by flow cytometry in NS and MF-stimulated AMs **(a)** Percentage of pHrodo-positive AMs at 2 and 8h post-infection. **(b)** Fold change of the percentage of pHrodo-positive cells from 2h to 8h. The dashed line represents the baseline (fold change = 1.0). Results are represented as min to max (Unpaired t-test with Welch’s correction, n=3). **(c)** Mean fluorescent intensity (MFI) of pHrodo-positive AMs at 2 and 8h post-infection. **(d)** Fold change of the MFI of pHrodo-positive cells from 2h to 8h. The dashed line represents the baseline (fold change = 1.0). Results are represented as min to max (Unpaired t-test with Welch’s correction, n=3).

Phagosome acidification was also assessed at 2h and 8h post-infection by flow cytometry using pHrodo-labelled *M. fortuitum*. The MF-stimulated group exhibited a significantly higher proportion of pHrodo-positive (i.e. acidified) cells at 8h post-infection (**Figures 5a, b**). The mean fluorescence intensity (MFI) of the pHrodo signal within pHrodo-positive cells increased slightly over time in both groups (**Figure 5c**), with a modest, non-statistically significant trend toward a higher increase at 8h post-infection in the MF–stimulated group (**Figure 5d**).

## 4 Discussion

This study provides an initial approach to an *in vitro* model of trained immunity, aimed at characterising the ability of *M. fortuitum* to prime caprine AMs towards a pro-inflammatory phenotype, potentiate cell activation and enhance antimicrobial activity upon homologous re-stimulation.

Given that AMs represent the principal innate immune barrier against respiratory mycobacteria, trained immunity modifications may offer broad-spectrum protection against zoonotic diseases such as TB (Russell et al., 2025; Mai et al., 2024; Cohen et al., 2018). Understanding how immune-boosting agents affect cell reprogramming is crucial for improving disease outcomes and reducing the economic and health burden of these infections.

Studies in the field have demonstrated that various species, including heat-killed *M. tuberculosis* (Minute et al., 2024), heat-inactivated *M. bovis* (Vaz-Rodrigues et al., 2022), Borrelia burgdorferi (Barriales et al., 2021) and the non-tuberculous mycobacteria *M. manresensis* (de Homdedeu et al., 2023), can induce trained immunity in different *in vitro* and *in vivo* settings. We chose *M. fortuitum* as the priming strain based on its rapid growth and biosecurity characteristics, which facilitate experimental workflows in short *in vitro* settings. Few studies have focused on goats, despite their importance as reservoirs for mycobacteria (Napp et al., 2013; Sweeney et al., 2012). In particular, due to the relevance of animal and human TB, the caprine model provides a practical framework for studying ruminant lung immunity markers, with findings that hold translational relevance for both cattle and humans (Cuenca-Lara et al., 2026).

Following primary MF-stimulation, a significant increase of all cytokines tested was observed, consistent with an initial pro-inflammatory response, as similarly detected by our previous results on stimulation of caprine AMs with different immunostimulats (Blay-Benach et al., 2026). Upon re-stimulation with MF, TNFα and IL-1β exhibited significantly increased responses compared to NS controls that only received the second MF stimulation, indicating the potential of MF as an inducer of a functionally pro-inflammatory trained immunity phenotype *in vitro*. Moreover, when comparing primary MF stimulation to re-stimulation (MF-MF), TNFα and IL-1β production was further elevated following re-stimulation, suggesting a trained immunity-associated effect. In contrast, IL-6 cells did not respond to the secondary stimulation. These cytokines have all been described as good indicators of trained immunity in both *in vitro* and *ex vivo* experiments. Similar results have been observed in human monocytes and macrophages trained with either β-glucan or BCG and re-stimulated with LPS (Bekkering et al., 2016; Helder et al., 2025). Furthermore, murine alveolar macrophages trained with LPS showed increased IL-6 production upon exposure to heat-inactivated *Streptococcus pneumoniae* (Zahalka et al., 2022). In the bovine model, both *in vitro* and *ex vivo* BCG training increased the production of these pro-inflammatory cytokines in exposed blood monocytes; however, no such increase was detected in vaccinated cows’ AMs (Guerra-Maupome et al., 2019).

Interestingly, IL-6 displayed a divergent response pattern following MF re-restimulation, showing a decline in its production at 96h compared to 24h, but with no further increase observed after re-stimulation. This could be correlated with the different post-transcriptional mechanisms that regulate each cytokine (Kadiyala et al., 2026). An increase was expected in IL-6 after re-stimulation in the MF-MF group, as observed in the other two cytokines; however, stimulation seemed to establish compensatory mechanisms that impede an elevated response against re-exposure. Although not experimentally assessed in this study, possible explanations for this divergent IL-6 response may include the post-transcriptional regulatory mechanism MK2-mediated mRNA stabilisation, whose deficiency has been shown to increase IL-6 mRNA degradation in stimulated macrophages. At the secretory level, the anti-inflammatory cytokine IL-10 can also act as an immune regulator on macrophages, inhibiting the production of different pro-inflammatory cytokines (Neininger et al., 2002; Tanaka et al., 2016; Rutz and Ouyang, 2016; Hirano, 2021). It is important to consider that the nature of the priming agent and the re-stimulation scheme may also influence which epigenetic and metabolic modifications are established and which responses are obtained (Kumar et al., 2024; Benjaskulluecha et al., 2022). Together with the duration of the resting period, these factors may shape macrophage responsiveness at each specific time point, as reflected by the failure of MF-cells to increase IL-6 production even upon MF re-stimulation.

Alongside pro-inflammatory cytokine production, additional markers are associated with stimulus-induced functional reprogramming of AMs. Although iNOS has not typically been considered a specific marker of trained immunity, its expression represents a relevant functional readout linked to enhanced bactericidal capacity and activation of the innate immune system (Mata et al., 2021; Rutschmann et al., 2022; Kang et al., 2025; Braverman and Stanley, 2017). Here, we provide a first assessment of iNOS production in caprine AMs as a potential marker of functional trained immunity-associated reprogramming induced by mycobacterial stimulus.

The increase in iNOS-producing cell subsets following MF re-stimulation in previously MF-stimulated cells, compared to NS controls, indicates a successful change of AMs towards an activated, bactericidal phenotype. iNOS is an important hallmark of pro-inflammatory macrophages due to its role in producing nitric oxide (NO), a key host defence molecule in immune response regulation (Bogdan et al., 2000; Xue et al., 2018). These results suggest a more robust NO-mediated response in animals previously exposed to *M. fortuitum*. Earlier studies in mice have shown that iNOS absence increased the risk of mycobacterial dissemination and growth in the lung (Adams et al., 1997; MacMicking et al., 1997). Regulation of NO production is necessary to prevent excessive inflammation during infection. Studies in mice have further demonstrated that iNOS promotes macrophage bactericidal activity against *M. tuberculosis* via the transcription factor Hypoxia inducible factor-1α (HIF-1α) while simultaneously limiting inflammation through the inhibition of the nuclear factor kappa-B (NF-κB) pathway (Braverman and Stanley, 2017).

Moreover, iNOS upregulation is closely associated with pro-inflammatory cytokine production in macrophages, as both processes are regulated by shared key transcriptional pathways (Xue et al., 2018; Kang et al., 2025; Salim et al., 2016). Accordingly, TNFα production was enhanced following MF re-stimulation in MF-primed AMs, coinciding with an increased frequency of iNOS-producing cells. This observation aligns with previous reports linking TNFα upregulation to NO production (Jacobs et al., 1996). Coordination between cytokine production and iNOS expression promotes an antimicrobial environment that contributes to host protection while modulating inflammation.

Finally, we sought to determine whether the pro-inflammatory and activated profile of AMs induced by MF stimulation translated into a sustained phagosomal acidification upon homologous challenge with *M. fortuitum*. From 24h after pHrodo-labelled *M. fortuitum* exposure onward, MF-trained AMs showed greater and sustained acidification over time. Of note, since bacteria were not removed from the culture throughout the 48h time-lapse, both stimulated and non-stimulated AMs had continued opportunity for phagocytosis; therefore, sustained pHrodo signal may reflect not only prolonged phagolysosome acidification but also ongoing bacterial uptake. Together, these observations may reflect the intracellular phagocytic capacity of the MF-trained cells.

Additionally, flow cytometry analysis revealed no differences in the proportion of pHrodo-positive cells at 2h post-infection, suggesting a similar *M. fortuitum* engulfment in both groups. However, at 8h post-infection, the proportion of pHrodo-positive cells was significantly higher in MF-trained AMs. This complementary approach for assessing phagosome maturation indicated not only more persistent acidification over time in MF-trained AMs, consistent with live-cell imaging results, but also a greater number of activated cells contributing to mycobacterial phagocytosis at early stages. MF-trained AMs display a predominant pro-inflammatory and antimycobacterial phenotype determined by pro-inflammatory cytokines and iNOS production and exhibit maintenance of acidification and improvement of early phagocytosis. Further functional assays are required to determine whether this more intense and prolonged acidification in MF-trained AMs is associated with increased bacterial lysis.

The presented results on live-cell imaging slightly differ from those we previously published in LPS-stimulated AMs (Blay-Benach et al., 2026), where a steeper acidification slope was found in LPS-stimulated compared to NS cells following *M. fortuitum* challenge. Several methodological differences may account for this discrepancy. In the present study, freshly obtained primary AMs were subjected to a trained immunity protocol that resembles the pipeline of innate immune memory *in vivo*, whereas previous work employed thawed cells, stimulation and immediate challenge. Furthermore, phagocytic activity was also assessed in this study by flow cytometry using pHrodo, which specifically reports phagosome acidification at earlier time points, in contrast to the prior approach, which inferred phagocytic efficiency indirectly from bacterial counts in cell lysates at 72 hours post-infection. This study represents an approach of homologous re-stimulation regimens and was conducted using AMs derived from a single goat. Therefore, these findings should be interpreted as proof of concept rather than a representation of population-level variability. Future studies should incorporate heterologous re-stimulation schemes with different resting periods to ensure no residual inflammation and validate the results across AMs from additional animals to strengthen the observed effects.

Previous studies employing murine models have also demonstrated improved phagocytosis using different trained immunity strategies *in vivo* and *ex vivo* (Zahalka et al., 2022; Chakraborty et al., 2023; Yao et al., 2018). Notably, the presented protocol successfully elicited an elevated pro-inflammatory profile and improved phagocytic activity associated with innate immune training *in vitro* in primary AMs, laying the groundwork for future *in vivo* studies in the caprine model, since the *in vitro* protocol cannot capture the systemic and mucosal complexity of the host. As previous work has shown divergent outcomes between *in vivo* and *ex vivo* approaches (Guerra-Maupome et al., 2019), validation in animal models or field studies will be needed to establish the translational potential of *M. fortuitum* in inducing functional reprogramming. Such studies would advance our understanding of vaccine-induced trained immunity in the lung mucosa and the efficacy of *in vivo* epigenetic reprogramming upon mycobacterial challenge.

## Data Availability

The raw data supporting the conclusions of this article will be made available by the authors, without undue reservation.
